# Antibacterial Potential of Bacterial Cellulose Impregnated with Green Synthesized Silver Nanoparticle Against *S. aureus* and *P. aeruginosa*

**DOI:** 10.1007/s00284-023-03182-7

**Published:** 2023-01-17

**Authors:** Mohamed T. Shaaban, Muhammad Zayed, Hussein S. Salama

**Affiliations:** Botany and Microbiology Department, Faculty of Science, Menoufia University, Shebin El-Kom, Egypt

## Abstract

In this study, bacterial cellulose (BC) impregnated with green synthesized silver nanoparticles (AgNPs) is evaluated as an antimicrobial membrane for wound-healing treatment. Green synthesized silver nanoparticles using *Moringa oleifera* leaf extract were characterized using UV‒visible spectroscopy, FTIR, X-ray diffraction, and transmission electron microscopy. The results confirmed that the resulted particles were Ag_2_O and metallic Ag in nanoscale with an average size ranged from 24 to 40 nm. The green synthesized nanoparticles incorporated within both bacterial cellulose and filter paper discs showed excellent antibacterial activities against *Staphylococcus aureus* ATCC 6538 and *Pseudomonas aeruginosa* ATCC 9027. There was no significant difference noticed between bacterial cellulose and filter paper holding capacity to nanoparticles and there was lack of interaction between bacterial cellulose and impregnated nanoparticles as elaborated by Fourier transform infrared spectral analyses. Scanning electron microscopy investigation showed major distortions effects of green synthesized silver nanoparticles on bacterial cell morphology.

**Supplementary Information:**

The online version contains supplementary material available at 10.1007/s00284-023-03182-7.

## Introduction

Bacterial cellulose (BC) is considered the most abundant hydrocolloid polymer suitable for use as a wound dressing [1] because it has a high water-holding capacity to absorb wound exudates, high mechanical strength to provide mechanical protection and a good physical barrier for wounds, high flexibility for wound healing, a 3-D network that facilitates the incorporation of various antimicrobial agents within it and good biocompatibility, which prevents inflammation and any adverse effects on skin and wounds [2].

Alteration in skin integrity caused by various factors such as an accident, surgical operations, or burns, increases the chance of wound infection by various pathogenic bacteria, which cause hazardous chronic inflammation throughout the wound healing process [3]. The global wound dressing market generated $11.4 billion revenue in 2017 and is projected to witness a CAGR of 7.2% during 2018–2023. Currently, more than 3000 types of wound healing dressings are available in the market but still there is no superior product that heals chronic wounds like diabetic wound, leg ulcers and pressure ulcers [4]. As a result, it is critical to protect these wounds from bacterial infection and inflammation using bacterial cellulose as a wound healing dressing.

The resistance of many pathogenic bacteria to antibiotics has increased rapidly during the last decades [5, 6]. Thus, it is important to custom an alternative antibacterial agent to protect wounds from bacterial infection and so the resulted hazardous chronic inflammation throughout the wound healing process. Currently, much research focuses on nanotechnology science to produce nanoparticles with effective antibacterial activity [7].

The matter is considered at the nanoscale when its size ranges from 1 to 100 nm [8]**.** Nanoparticles have a large surface area due to their nanoscale size, which contributes to their enhanced physical and chemical properties, which are useful in a variety of fields such as antimicrobial properties [9]. Traditional methods for the synthesis of nanoparticles as physical and chemical methods have limited use due to toxic chemicals, required high-energy input and costly downstream processing are high cost [7]. There are different biological methods for the green synthesis of nanoparticles using microorganisms, enzymes, and plant extracts, which have been suggested as possible eco-friendly, less toxic, high yield, rapid synthesis and low-cost downstream processes. The use of plant extracts for the green synthesis of silver nanoparticles involves a chemical reaction between phytocompounds present in the plant extract and silver nitrate [10, 11].

*Moringa oleifera* plant parts are used for medicinal purposes as it possesses antiatherosclerosis and antioxidant effects. The major leaves constituents are phenolic compounds and flavonoids such as cryptochlorogenic acid, isoquercetin and astragalin which are famous for their wide-ranged activities including antioxidation, antihypertension and antiinflammation [11, 12].

This research focuses on the incorporation of green synthesized silver nanoparticles within bacterial cellulose and evaluation their antibacterial activity for future use of this composite as a wound healing dressing.

## Materials and Methods

### Green Synthesis of Silver Nanoparticles (AgNPs)

Leaves of *Moringa oleifera* were collected, washed three times with distilled sterilized water, and dried at 50 °C in an oven. Then, 10 g of dried leaf powder was added to 100 mL of deionized water in a 500 mL Erlenmeyer flask, boiled for 20 min, and then filtered. The filtrate (leaf extract) obtained was stored at 4 °C for further use.

For green synthesis of silver nanoparticles, 10 mL of leaf extract was mixed with 90 mL of 1 mM aqueous AgNO_3_ and heated at 60 °C for 20 min. A change from brown to reddish-brown color was observed, which indicates AgNPs formation [12].

### Purification and Determination of AgNPs Concentration

The produced AgNPs suspension was split into ten pre-weighed sterilized Falcon tubes, 10 mL in each tube. The suspension was then centrifuged at 4000 rpm for 2 h at 4 °C. The supernatants were discarded, and the precipitates were washed with 10 mL distilled sterilized water to remove any contaminating plant materials and then centrifuged again at 4000 rpm for 2 h at 4 °C. The washing step was repeated three times. After washing and centrifugation, the five tubes were dried at 37 °C for 24 h and weighed. Finally, the precipitates within remained five tubes were resuspended in 1 mL sterilized distilled water for characterization and antimicrobial activity tests.

### Characterization of Green Synthesized AgNPs

#### Ultraviolet Visible (UV‒Vis) Spectral Analyses

The UV‒visible absorption spectra for the AgNPs suspension were recorded in a quartz cuvette at wavelengths ranging from 300 to 1100 nm using a UV‒vis–NIR spectrometer (JASCOV-570, China).

#### X-ray Diffraction (XRD) Analyses

The structure and crystallinity of the green synthesized AgNPs were analyzed using an XRD apparatus equipped (Bruker D2 Phaser 2nd Gen, American) with a Cu (Ka) (1.5406 Å) radiation source. XRD spectra were recorded over 2θ angles ranging from 10° to 60°. The average crystallite size of the nanoparticles was calculated from the XRD pattern using the Deby‐Seherrer formula *D* = 0.9λ/ β Cosθ, where *λ* is the wavelength of the X-rays used for diffraction and *β* is full width at half maximum (FWHM) of a peak [13].

The value of d- spacing has been calculated using Bragg’s Law, 2dSinθ = nλ, where n is the order of diffraction pattern. n is equal to 1. To estimate FWHM, observed peaks were fitted with a Gaussian function using origin 8.5. [14].

#### Transmission Electron Microscopy (TEM) of AgNPs

The size and shape of the synthesized AgNPs were estimated using a transmission electron microscope (model JEOL-1230, Japan). After biosynthesis and washing, 10 µL of AgNPs suspension was loaded onto a clean copper grid coated with carbon, and the size was determined after examination under a microscope.

### Antimicrobial Activity Assay

*Staphylococcus aureus* ATCC 6538 and *Pseudomonas aeruginosa* ATCC 9027, were used to screen the inhibitory effects of green synthesized AgNPs. This assay was performed in triplicate by pouring 20 mL of sterilized Mueller–Hinton agar medium into sterilized Petri dishes and allowed to solidify under aseptic conditions. Then, 100 μL of standardized inoculum (0.5 McFarland) were uniformly spread on the surface of the plate. Then, sterilized filter paper discs (Whatman filter paper 6 mm diameter) saturated with 20 μL of green synthesized AgNPs suspension were applied to the surface of the seeded agar plates. Sterilized filter paper discs saturated with 20 μL of distilled sterilized water were used as a negative control. The plates were kept at 4 °C for 2 h to allow equal dispersion and incubated at 37 °C for 24 h. The antimicrobial activity was evaluated by observing the inhibition zone diameter **(**IZD**)** around the discs [15]**.**

### Minimal Inhibitory Concentration and Minimal Bactericidal Concentration

The minimal inhibitory concentrations (MICs) of AgNPs were evaluated by broth microdilution methods using 96-well microtiter plates according to CLSI (2012) guidelines with some modifications [16, 17]. Twofold dilutions of nanoparticle suspensions were made to make final concentrations of 2.5 to 0.0012 mg/mL after adding 0.1 mL to each well, which was previously supplemented by 0.1 mL Mueller Hinton broth media. Then, 10 µL of standard inoculum was inoculated into each well. After incubation at 37 °C for 24 h, 30 µL of resazurin solution (0.18% w/v) was added to each well, and the plates were re-incubated at 37 °C for another 24 h. The MIC was recorded as the lowest concentration without bacterial growth (no color changes in resazurin solution). Media inoculated with bacteria were used as a positive control. Only media and media plus AgNPs were used as negative controls.

The minimal bactericidal concentrations (MBCs) were determined by transferring 30 µL from each well to Mueller Hinton agar medium and incubating at 37 °C for 24 h. The lowest concentration without apparent microbial growth was recorded as the MBC [16].

### Production and Preparation of Bacterial Cellulose

For the production of bacterial cellulose, 50 mL of sterilized modified GAM medium [18] was inoculated with 1 mL of *Novacetimonas hansenii* HS1 culture and incubated for 7 days at 30 °C. The pellicles produced after incubation were purified by treatment with 0.5 M NaOH at 90 °C for 1 h to eliminate attached cells, followed by three washes with distilled water and drying at 60 °C. Then, the purified BC films were cut into 6 mm discs, sterilized in distilled water at 121 °C for 20 min, and dried for further use [19].

### Incorporation of AgNPs Within Bacterial Cellulose and Filter Paper

Sterilized dried bacterial cellulose (BC) and filter paper (FP) discs were immersed in 0.2 mL AgNPs suspensions of different concentrations (5, 2.5, and 1.25 µL/mL) for 24 h [19]. After impregnation, the antimicrobial activities of AgNPs were estimated using the disc diffusion method. Pure sterilized BC and FP discs saturated with 0.2 mL distilled sterilized water were used as a negative control.

### Calculation of Bacterial Cellulose and Filter Paper Holding Capacities to AgNPs

Holding capacities of bacterial cellulose and filter paper impregnated with AgNPs were calculated according to [19]. The pre-weighed impregnated sterilized bacterial cellulose and filter paper discs with silver nanoparticles were centrifuged at 2000 rpm for 5 min and weighed, then discs centrifuged at 2000 rpm for 5 min and weighed. Finally, discs centrifuged at 2000 rpm for 5 min and weighed. Separately, holding capacities of BC or FP were calculated every 5 min of centrifugation according to the following equation:

[Holding capacity (%) = (W_h_–W_d_)/W_d_ × 100, where W_h_ is the weight of the disc after centrifugation and W_d_ is the dry weight of the BC and FP discs; each measurement was carried out in triplicate.

### Fourier Transform Infrared (FTIR) Spectroscopy Analyses

Dried AgNPs, dried purified bacterial cellulose, and bacterial cellulose incorporated with AgNPs were analyzed using an FTIR spectrometer [20], and spectra were collected at wavenumber from 400 to 4000 cm^−1^ on a VERTEX 80v spectrometer (Bruker, 4 cm^−1^, 128 scans, American).

### Scanning Electron Microscopy (SEM) Investigation of Bacterial Cells

Samples were prepared for SEM investigation according to [21] with some modifications. Ten millimeters of sterilized nutrient broth containing MIC of AgNPs were inoculated by 1 mL bacterial stock culture (approximately 10^7^ CFU/mL) and incubated at 37 °C for 18 h. After incubation, 900 µL of bacterial culture was transferred into Eppendorf tubes for sample treatment as well as for control and the cultures were centrifuged at 3000 rpm for 5 min. After centrifugation the supernatant was discarded, and the pellet washed three times using 1 mL of phosphate buffer solution (PBS) 0.05 M (pH 7.4). After washing the pellets were resuspended in fixative solution till analyses.

For SEM investigation, bacterial cells were prepared and coated with gold using an ion sputter instrument and analyzed using a scanning electron microscope (JEOL JSM- IT200, Japan) to investigate the effect of green synthesized AgNPs on bacterial cell morphology.

### Statistical Analyses

All experiments were done in triplicate, using the R language [22]. The results were expressed as the mean values±standard deviations, the adjusted p values were calculated in R using Tukey’s test, and significance was considered when the adjusted p value was equal to or less than 0.05 between the compared treatments. Then, graphs were presented using the ggplot2 package in R [23].

## Results

The green synthesized AgNPs concentration estimation after biosynthesis showed that the mixture (10 mL leaf extract+90 mL of 1 mM AgNO_3_) produced 25±0.305 mg silver nanoparticles. After that the green synthesized nanoparticles exhibit characterization by UV–Vis spectroscopy, XRD, FTIR, and TEM.

**Fig. 1:**
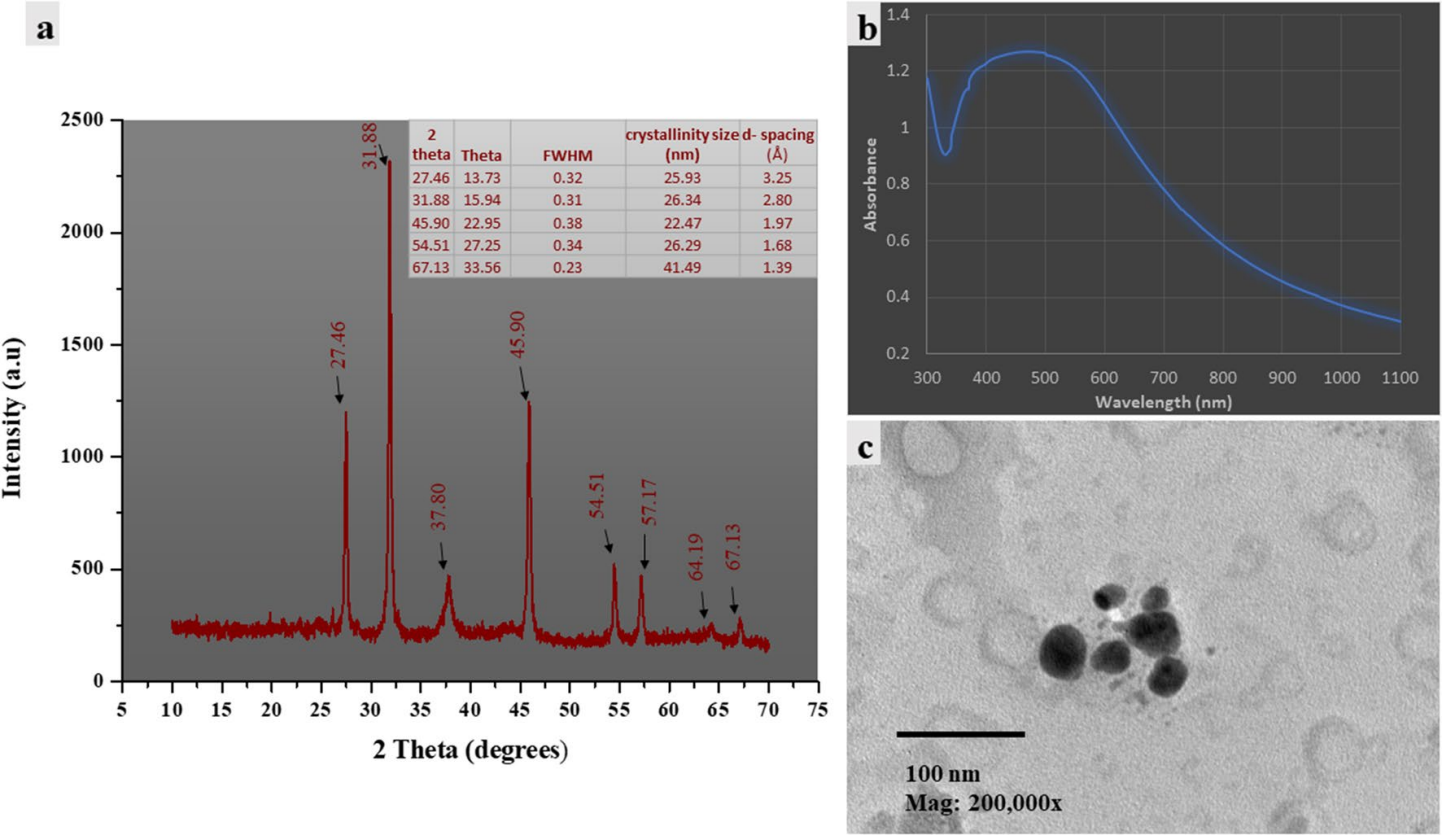
Characterization of green synthesized nanoparticles **a:** X-ray diffraction analyses of green synthesized silver nanoparticles. XRD spectra were recorded over 2θ angles ranging from 10° to 60°; **b:** ultraviolet visible spectrum analyses for green synthesized silver nanoparticles using *Moringa oleifera* leaves extract, the spectrum wavelength ranged from 300 to 1100 nm.; **c:** transmission electron microscopy investigation for silver oxide nanoparticles (Color figure online)

Characterization of green synthesized nanoparticles using UV–Vis spectroscopy showed the maximum absorption peak at 466 nm, and there was broadness in the resulting peak. As shown in (Fig. 1), the XRD pattern obtained for the sample showed intense diffraction bands at 27.46°, 31.88° and 45.90° and there are other diffraction peaks appeared at 54.51°, 57.17°,64.19°, and 67.13°. Transmission electron microscopy (TEM) investigation showed that the green synthesized silver nanoparticles were semispherical particles shapes with average particle sizes ranging from 24 to 40 nm. In FTIR spectrum of AgNPs, bands observed at 2935 and 2866 cm^−1^ region arising from C–H stretching of aromatic compound. The presence of bands at 1051 cm^−1^ indicated the presence ether linkages (C–O–C) functional groups. The band at 1631 cm^−1^ in the spectrum corresponds to C–N and C–C stretching. The band at 1468.53 cm^−1^ was assigned for N–H stretch vibration present in the amide linkages of the proteins  (Fig. 3).

**Fig. 2:**
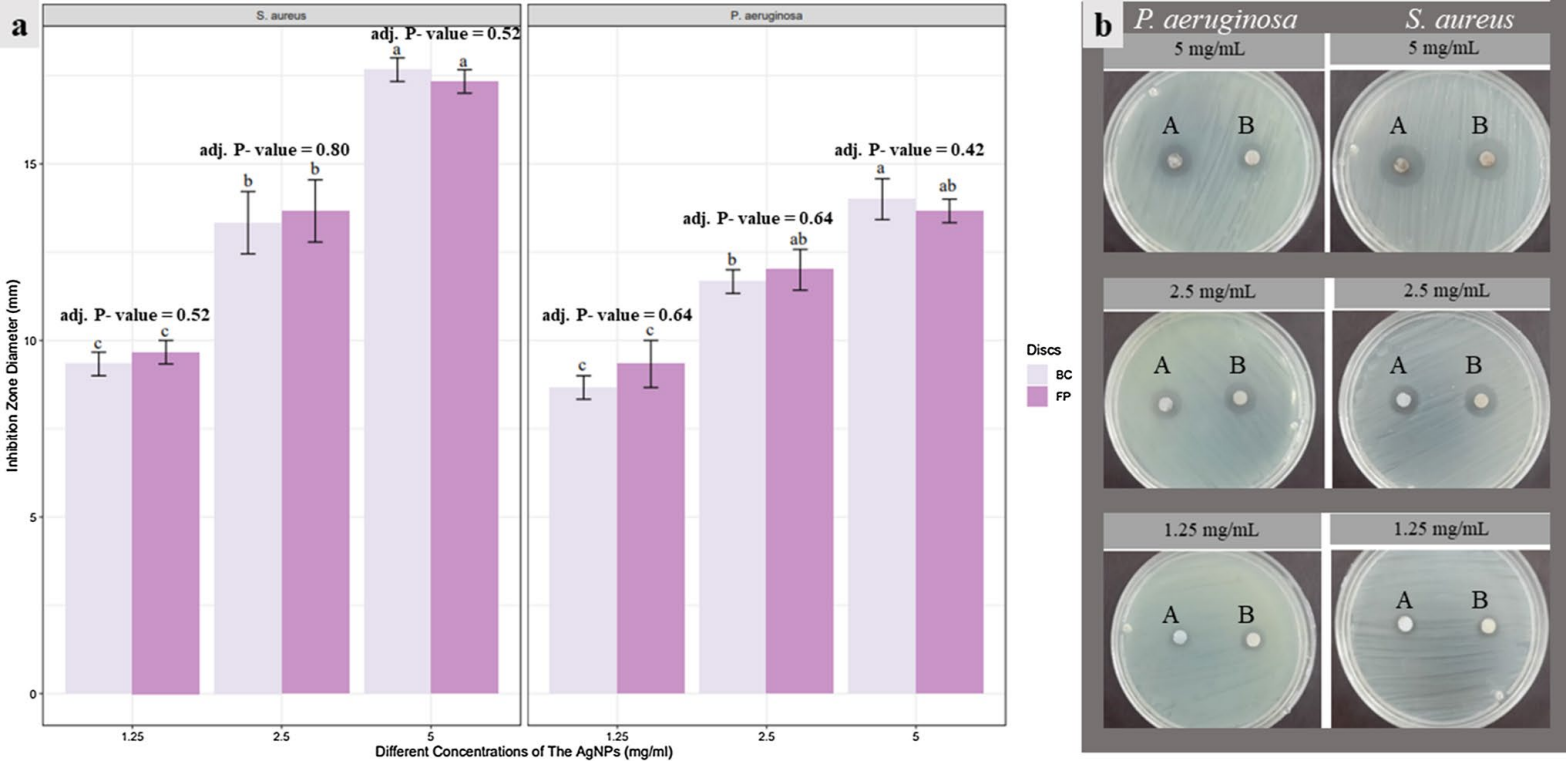
**a:** A comparison of antibacterial activities of different concentrations of green synthesized silver nanoparticles (5, 2.5, and 5 mg/mL) when loaded on filter paper discs versus bacterial cellulose discs against S. aureus and the P. aeruginosa as measured by IZD in millimeter. Bars represent the mean values of three green synthesized replicates±SD. The small alphabetical letters on the bars represent statistically significant differences between samples in each facet independently (adjusted *P*-value < 0.05 Tukey’s test); **b:** Antibacterial activities for these nanoparticles with different concentrations (5, 2.5, and 5 mg/mL), where the discs labeled with (A) on the left represents bacterial cellulose loaded with nanoparticles. However, the discs labeled with (B) on the right is for the filter paper disc loaded with the same concentration of nanoparticles (Color figure online)

 The antibacterial activity of the green synthesized AgNPs (5 mg/mL) was evaluated against *S. aureus* and *P. aeruginosa* using the disc diffusion method. The resulting inhibition zone diameters estimated in the case of *S. aureus* was 17.6±0.58 mm and that estimated in the case of *P. aeruginosa* was 12.6±0.58 mm (Fig. 2).

The results of the minimal inhibitory concentration assay indicated that the MIC of green synthesized AgNPs against *S. aureus* was 1.25 mg/mL and the MIC against *P. aeruginosa* was 1.25 mg/mL. The minimal bactericidal concentration (MBC) of green synthesized AgNPs against *S. aureus* was 1.25 mg/mL and against *P. aeruginosa* was 1.25 mg/mL.

Incorporation of green synthesized AgNPs within bacterial cellulose and filter paper discs as shown in indicated that both bacterial cellulose and filter paper discs incorporated with silver nanoparticles have excellent antibacterial activity against two tested bacterial strains, and there was no significant difference observed between the antibacterial activities of bacterial cellulose and filter paper incorporated with three concentrations of AgNPs when estimated against *S. aureus* and *P. aeruginosa*.

The values of holding capacity of bacterial cellulose and filter paper indicated that there was no significant difference between bacterial cellulose and filter paper holding capacity to green synthesized AgNPs as the adjusted p values estimated were more than 0.05.

**Fig. 3:**
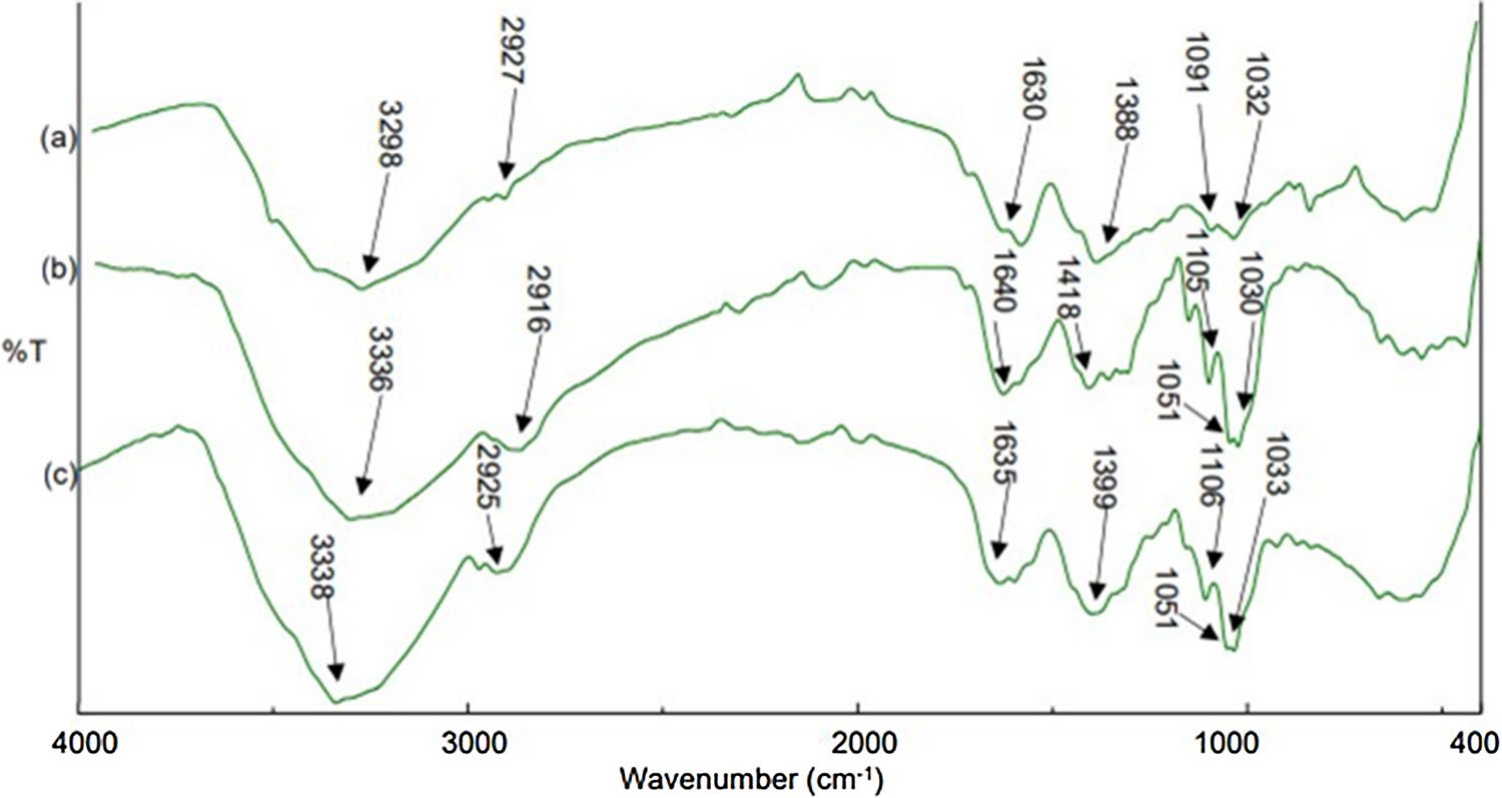
Fourier transform infrared analyses for **a**- Bacterial cellulose, **b**- Silver nanoparticles, and **c-** Bacterial cellulose incorporated with green synthesized silver nanoparticles. Spectra were collected at wavenumber from 400 to 4000 cm^−1^ (Color figure online)

As shown in (Fig. 3) Fourier transform infrared analyses was performed for AgNPs, purified bacterial cellulose and bacterial cellulose incorporated with AgNPs. The bands observed at 3298, 3336 and 3338 cm^−1^ correspond to O–H stretching, and the bands observed at 2916, 2925, and 2927 cm^−1^ indicate the presence of C–H stretching. The bands at 1630, 1632, and 1633 cm^−1^ were assigned to absorbed water. Additionally, the presence of bands at 1388, 1399, and 1418 cm^−1^ indicated the presence of C–H bending vibrations. The bands at 1091, 1105, and 1106 cm^−1^ were assigned to ether linkages (C–O–C) from glycosidic components. The bands at 1030, 1032, and 1033 indicate the presence of C–C, C–OH, C–H of ring, side group vibrations. The presence of bands at 1051 cm^−1^ in AgNPs and BC incorporated with AgNPs indicated the presence ether linkages and C–O or C–O–C functional groups.

**Fig. 4:**
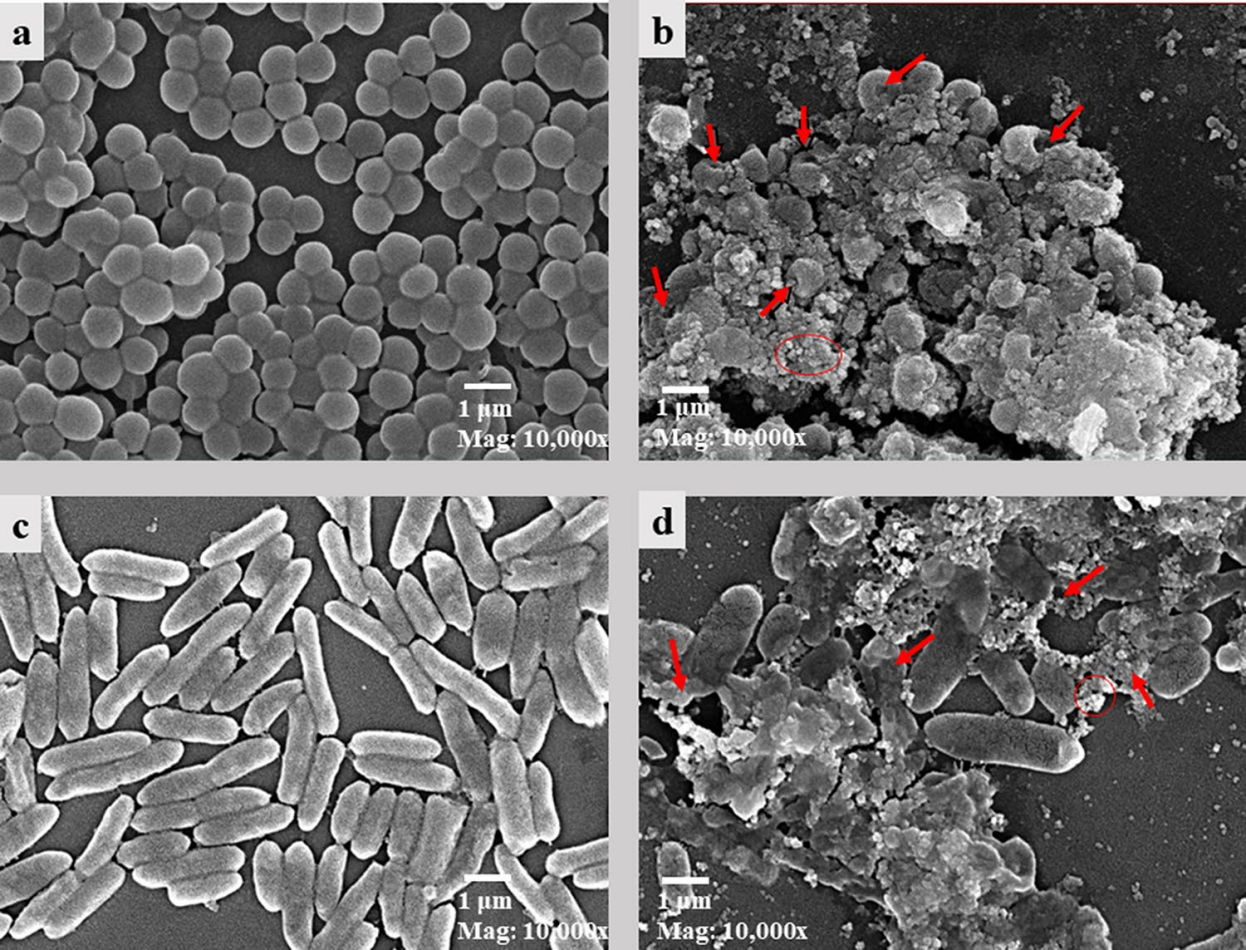
Scanning electron microscopy investigation for **a**- *S. aureus* (control); **b**- *S. aureus* (treated by the minimum inhibitory concentration of AgNPs); **c**- *P. aeruginosa* (control) and **d**- *P. aeruginosa* (treated by the minimum inhibitory concentration of AgNPs)*.* Red arrows in figure (**b**) and (**c**) pointed to distortions in bacterial cells and red circle put around accumulation of nanoparticles on bacterial cells (Color figure online)

Scanning electron microscopy showed great differences in cell morphology between control and treated cells with 1.25 mg/mL of green synthesized AgNPs. As seen in (Fig. 4), there was accumulation of AgNPs on *S. aureus* cells, which results in pores or invaginations on cells that affect bacterial viability. On the other hand, *P. aeruginosa* cells treated with AgNPs showed destruction and distortion at cell poles due to an accumulation of nanoparticles at cell poles that affected cell viability and caused cell death.

## Discussion

After green synthesis of silver nanoparticles using *Moringa oleifera* leaf extract, the solution color changed to reddish brown due to the surface plasmon resonance phenomenon, which in turn resulted in the intense absorption peak exhibition of silver nanoparticles. In the UV‒Vis spectrum the maximum peak intensity observed at 466 nm confirms the green synthesis of silver nanoparticles. These finding was is in agreement with data in the UV–Visible spectrum of [24, 25]. The broadness of the peak indicates the presence of polydispersed nanoparticles particles as discussed by Ibrahim et al. in (2021) [26] who attributed the broadness of absorption band due to an increasing of a particle size distribution.

In X-ray diffraction, the intense peaks observed at 27.46° and 31.88° corresponds to (110) and (111) of Ag_2_O. Diffraction peaks at 45.90°, 54.51° and 67.13° could be due to (211), (220) and (222) planes of face‐center cubic silver, respectively as compared with the standard Ag_2_O (JCPDS 76‐1393). Also, same results reported by [27–29]. Unknown peaks observed at 57.17°, 64.19° may correspond to any contamination from plant leaf extract.

The average size of green synthesized nanoparticles calculated using Deby‐Seherrer equation was 28.5 nm which was nearly in accordance with the results of transmission electron microscopy investigation which indicated that the size of green synthesized silver nanoparticles using *moringa oleifera* leaf extract ranged between 24 and 40 nm with semispherical shaped nanoparticles [25].

FTIR analyses of AgNPs indicated the presence of C–N and C–C stretching which in turn indicate the presence of proteins which also reported by [29, 30]. Also, detection of N–H stretch vibration indicates the presence of amide linkages of the proteins. The presence of ether linkages C–O–C confirming the presence of flavones, terpenoids and polysaccharides present in *Moringa oleifera* leaf extract which have good reduction potential involving in bio-reduction of AgNO_3_ as reported by Jadhav et al.(2022) and (Moodley et al. (2018)[11, 31]. Also, Kota et al.in (2017) [13] reported that the presence of reactive N–H and O–H groups that are effective in reducing Ag(I) ions to Ag (0) So, presence of flavones, terpenoids, polysaccharides, proteins and aromatic groups of polyphenols indicating the role of reduction potential of these compounds in bio-reduction, stability and capping of AgNPs,

Green synthesized silver nanoparticles showed good antibacterial activities against *S. aureus* and *P. aeruginosa*. Also, Prasad and Elumalai in (2011) and Ugwoke et al. in (2020) reported the excellent antibacterial activity of green synthesized AgNPs using *Moringa oleifera* leaf extract [12, 24]. So, ecofriendly green synthesized silver nanoparticles have an effective antibacterial activity against different bacterial strains.

The recorded minimum inhibitory concentrations (1.25 mg/mL) and minimum bactericidal concentrations (1.25 mg/mL) indicated that green synthesized AgNPs have a bactericidal effect against *S. aureus* and *P. aeruginosa*.

Incorporation of different concentrations of green synthesized AgNPs into bacterial cellulose and filter paper discs was performed, and their antibacterial activities were estimated in both cases. The results indicated that both BC- AgNPs composites and FP-AgNPs composites showed good antibacterial activities against *S. aureus* and *P. aeruginosa.* Barud et al. in (2011) [32] also reported the antibacterial efficiency of bacterial cellulose-silver nanoparticle composites against *S. aureus* and *P. aeruginosa*. Pal et al. in (2017) [33] reported a good antibacterial activity of the bacterial cellulose silver nanoparticles composites against *Escherichia coli.* Also, Ibrahim et al.in (2021) [26] showed excellent antibacterial activity of BC- AgNPs composite against *S. aureus* as Gram-positive bacteria and *E. coli* as Gram-negative bacteria.

The holding capacities results indicate there were no significant difference between BC and FP despite the materials properties of both being different. This is may be due to the lack of interaction between nanoparticles incorporated within BC and FP. These findings in turn recommend using BC-AgNPs as a wound healing dressing rather than using FP-AgNPs due to the advantages and biocompatibility of BC.

The appearance of all functional groups in FTIR analyses in our manuscript as reported by [7, 20, 25, 34] confirming the structure of bacterial cellulose. The presence of the same functional groups in bacterial cellulose, green synthesized AgNPs and BC incorporated with AgNPs, with some difference in transmittance intensities due to functional groups of AgNPs, indicating the absence of any chemical interaction and the nanocomposite may be formed due to the entrance of silver nanoparticles within the microporous network structure of BC. Similar results were also reported by Jinga et al. and Audtarat et al. This lack of interaction between nanoparticles and bacterial cellulose may facilitates the liberation of nanoparticles incorporated within BC, which in turn affects pathogenic bacteria. In green synthesized AgNPs and BC incorporated with AgNPs spectra, there were bands indicating the presence of ether linkages C–O–C [7], which are groups of flavones, terpenoids and polysaccharides present in *Moringa oleifera* leaf extract, involved in capping and stabilization of green synthesized AgNPs.

The effects of green synthesized AgNPs on bacterial cells morphology were investigated using SEM. The results showed great distortion effects of green synthesized AgNPs on cells morphology which indicate the efficiency of green synthesized AgNPs. Also, in case of *P. aeruginosa* there were great morphological distortion effects of the minimum inhibitory concentration of green synthesized AgNPs on bacterial cell.

## Conclusion

Characterization of green synthesized nanoparticles using UV-spectrum, FTIR, XRD, and TEM indicated that the produced nanoparticles were Ag_2_O with average size 32 nm. Incorporated green synthesized AgNPs within bacterial cellulose and filter paper discs showed excellent antibacterial activity against *S. aureus* and *P. aeruginosa*. Fourier transform infrared analyses confirmed no interaction between green synthesized AgNPs and bacterial cellulose and scanning electron microscopy investigation showed major distortion of the two tested bacterial strains treated by green synthesized AgNPs.

Finally, our findings of excellent antibacterial activity and same holding capacities of BC and FP to AgNPs recommend future using of biocompatible BC-AgNPs composite rather than using FP-AgNPs composite but further studies needed to estimate the efficiency of progressed applications of BC- AgNPs composite.

## Supplementary Information

Below is the link to the electronic supplementary material.

Supplementary file1 (TIFF 270 KB)

Supplementary file2 (TIF 711 KB)

## Data Availability

All data are available with corresponding author.
