# Supplementary figures and images for: Antibacterial Potential of Bacterial Cellulose Impregnated with Green Synthesized Silver Nanoparticle Against S. aureus and P. aeruginosa
Source: Curr Microbiol. 2023 Jan 17;80(2):75. doi: 10.1007/s00284-023-03182-7 (PMC9845145; doi:10.1007/s00284-023-03182-7)

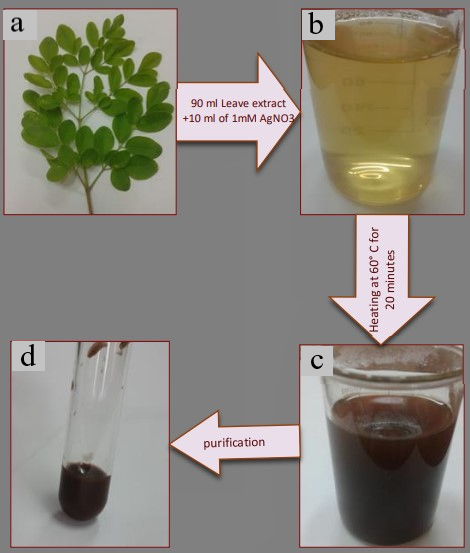

Supplement: Supplementary file 1 — Supplementary file1 (TIFF 270 KB) [file 284_2023_3182_MOESM1_ESM.tiff]

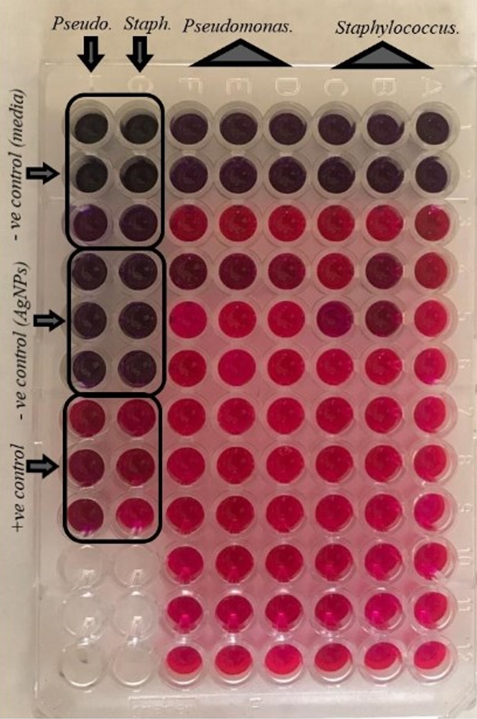

Supplement: Supplementary file 2 — Supplementary file2 (TIF 711 KB) [file 284_2023_3182_MOESM2_ESM.tif]
